# Manual compression versus MANTA device for access management after impella removal on the ICU

**DOI:** 10.1038/s41598-022-18184-x

**Published:** 2022-08-18

**Authors:** Florim Cuculi, Philipp Burkart, Giacomo Cioffi, Federico Moccetti, Mehdi Madanchi, Thomas Seiler, Stefanie Hess, Mathias Wolfrum, Magiliny Jeyarasa, Sonja Meier, Silvia Kuzmiakova, Maani Hakimi, Robert Seelos, Richard Kobza, Stefan Toggweiler, Adrian Attinger-Toller, Matthias Bossard

**Affiliations:** 1 Cardiology Division, Heart Center Lucerne, Luzerner Kantonsspital, 6000 Luzern 16, Switzerland; 2 Medical School, University of Zurich, Zurich, Switzerland; 3 Division of Vascular Surgery, Luzerner Kantonsspital, Lucerne, Switzerland

**Keywords:** Interventional cardiology, Medical research, Outcomes research

## Abstract

To compare the safety and efficacy of manual compression versus use of the MANTA closure device for access management after Impella removal on the intensive care unit (ICU). The number of patients treated with percutaneous left ventricular assist devices (pLVAD), namely Impella and ECMO, for complex cardiac procedures or shock, is growing. However, removal of pLVAD and large bore arteriotomy closure among such patients on the ICU remains challenging, since it is associated with a high risk for bleeding and vascular complications. Patients included in a prospective registry between 2017 and 2020 were analyzed. Bleeding and vascular access site complications were assessed and adjudicated according to VARC-2 criteria. We analyzed a cohort of 87 consecutive patients, who underwent access closure after Impella removal on ICU by using either the MANTA device or manual compression. The cohort´s mean age was 66.1 ± 10.7 years and 76 patients (87%) were recovering from CS. Mean support time was 40 h (interquartile range 24–69 h). MANTA was used in 31 patients (35.6%) and manual compression was applied in 56 patients (64.4%). Overall access related bleedings were significantly lower in the MANTA group (6.5% versus 39.3% (odds ratio (OR) 0.10, 95% CI 0.01–0.50; *p* = 0.001), and there was no significant difference in vascular complications between the two groups (*p* = 0.55). Our data suggests that the application of the MANTA device directly on the ICU is safe. In addition, it seems to reduce access related bleeding without increasing the risk of vascular complications.

## Introduction

During the last decade, the number of procedures and devices using large bore femoral vascular access has steadily grown^1^. Such procedures include for instance transcatheter aortic valve replacement (TAVR), endovascular aneurysm repair (EVAR), but also percutaneous left ventricular assist devices (pLVAD). In this context, the catheter-based, continuous axial flow pumps Impella 2.5 and CP have become the preferred pLVADs for patients with profound cardiogenic shock (CS) or requiring high-risk percutaneous coronary interventions (PCI)^2^.

Although, there is mounting data about strategies and devices for vascular closure in patients undergoing TAVR and EVAR, studies focusing on optimal non-surgical access closure in patients managed with the pLAVD Impella remain scarce. In this context, one needs to take into account that Impella removal on the intensive care unit (ICU) is particularly challenging and is prone to bleeding and vascular complications^3–5^. Manual compression is often standard of care, but this is time-consuming and frequently semi-effective^5^.

For TAVR and EVAR, the MANTA device, which represents a collagen-based, vascular closure device specifically designed for large bore femoral access site closure (12 to 24 French), truly facilitates access management, as highlighted by a series of recent studies^6–8^. However, the utility and safety of this vascular closure device has not been systematically evaluated in patients requiring pLVAD support with an Impella. Especially patients, which require prolonged mechanical support and are transferred to the ICU after Impella insertion in the catheter laboratory, might benefit from a device facilitating access closure while recovering.

Therefore, the aim of this analysis is to report the safety and efficacy of the MANTA device compared to manual compression among patients, which undergo Impella explantation while residing on the ICU.

## Methods

### Study population

From a prospective registry, we analyzed consecutive patients in cardiogenic shock (CS) or undergoing high-risk PCI, who required prolonged hemodynamic circulatory support by Impella 2.5, CP or CP Smart Assist (Abiomed Inc., Aachen, Germany) at the Heart Center of the Luzerner Kantonsspital between January 2017 and December 2020. The study conduct complies with the Declaration of Helsinki. Prospective data acquisition after enrollment was approved by the local and national ethics committee (*EKNZ/Swissethics*, BASEC-ID 2019-00274, *ClinicalTrials.gov Identifier*: NCT04117230). Patients or their relatives provided written informed consent.

### Impella device

The catheter-based, continuous microaxial flow pumps Impella 2.5, CP and CP Smart Assist can be implanted fully percutaneously, generally through the femoral artery^2,5^. The Impella 2.5 and CP have an introducer sheath diameter of 12 and 14 French, respectively^2,5^. As part of our clinical routine, we obtained a contrast angiogram in an ipsilateral projection to assess puncture height and anatomical suitability of the iliac and femoral arteries prior to Impella insertion. Ultrasound guidance was used, whenever possible. However, considering the emergency setting of device implantation in CS patients ultrasound guidance was left to discretion of the treating physician. All patients were anticoagulated with unfractionated heparin (UFH) to achieve an activated clotting time (ACT) > 250 s during PCI. Finally, the Impella devices were inserted over a stiff 0.018-inch guide wire^2,5,9^, advanced under fluoroscopy and positioned in a retrograde fashion across the aortic valve.

### Access closure strategies on ICU

For access management after transfemoral implantation of an Impella device, the following strategies for vascular closure on the ICU were utilized at our institution: (I) manual compression, (II) access closure using MANTA 14F device, or (III) surgical removal in selected cases at high risk for bleeding or ischemic complications. Of note, pre-closure using the Perclose ProGlide device (Abbott Vascular Inc., Santa Clara CA, U.S.A.) might only be applied in elective cases requiring short-term hemodynamic support at our institution, due to concerns about access site infections while leaving the sutures exposed on the ICU.

Our standard operating procedure for manual compression after transfemoral Impella device removal recommends withholding heparin for at least 2 h and until the ACT drops < 150 s. Then, we applied manual compression for 10–20 min, followed by mechanical compression using the inflatable FemoStop device (Abbott Vascular Inc., Santa Clara CA, U.S.A.) if needed, which allows gradual pressure release for 120–240 min.

The MANTA device (Teleflex Inc., Morrisville, North Carolina) is a dedicated large-bore arteriotomy closure device, which may be used for access diameters ranging from 12-Fr to 25-F in outer diameter. Two sizes are available (14- and 18-Fr)^6–8^. It comprises of a luminal resorbable polymer toggle and an extraluminal, resorbable, bovine collagen hemostatic plug. The toggle and plug are pulled together with a non-resorbable polyester suture topped by a radiopaque, stainless steel suture lock. We started using the MANTA device in March 2018 and all operators have extensive experience, with its deployment (including at least 10 supervised cases)^7^.

In cases of (I) low puncture height (e.g. below the femoral bifurcation), (II) small vascular diameter (< 6 mm by visual estimation), and/or (III) prohibitive peripheral arterial disease (PAD), we did not deploy the MANTA device^7^. Before access closure with the MANTA device, we aimed for an ACT < 180 s. After extensive local disinfection sterile drapes were used to create a similar environment as in the cardiac catheterization laboratory. Over the Impella introducer sheath side port, we inserted a 0.035-inch guidewire, measured arterial access depth using the dedicated puncture depth locator and deployed the device. More details about the MANTA device deployment can be found elsewhere^6^. Manual compression or pressure bandages were only applied in cases if immediate hemostasis was not achieved.

### Data collection, outcomes and definitions

Baseline demographics, procedural characteristics and outcome data were collected by study personal and entered in a dedicated database. All baseline angiograms of the iliac and femoral arteries were analyzed by a trained physician (P.B.) and quality of measurements was reviewed by a senior physician (A.T.). The minimal luminal diameter, degree of stenosis, severity of calcification, or other vascular abnormalities were determined. Measurement of puncture height was related to the center of the femoral head.

For the purpose of this study, we defined ‘immediate hemostasis’ as no relevant bleeding or additional medical measures (e.g. additional manual compression) directly after access closure or release of manual/mechanical compression. Strategy success was achieved when there were no signs of bleeding within 30 min after strategy termination. In case of MANTA usage strategy success further included correct device deployment and no function failure of the device. Procedural and access site–related complications were assessed according to the Valve Academic Research Consortium (VARC)–2 criteria and adjudicated by two independent investigators (T.S. and M.M.)^10^. In case of disagreement, consensus was reached through discussion with a senior physician (A.T.). Additionally, the following clinical outcomes within 30 days after the index event were analyzed: (I) minor or major bleeding according to the Bleeding Academic Research Consortium (BARC) criteria, (II) new myocardial infarction (MI), (III) stroke/transient ischemic attack (TIA), (IV) repeat cardiogenic shock, (V) cardiovascular death and all-cause death^11^.

### Statistical analysis

Continuous data are presented as mean ± standard deviation (SD) or median (interquartile range (IQR)) as appropriate. Categorical variables are displayed as numbers (percentage). For comparison of variables the odds ratio (OR) and the 95% confidence interval (CI) were calculated. Furthermore, continuous variables were compared using the student’s t-test or the Wilcoxon rank sum test. For statistical comparison between groups the Man-Whitney-U test, chi-square test, Fishers exact test or students t-test were used. Categorical variables were compared using the chi-square test or the Fisher’s exact test as appropriate. For the 30-day follow-up cox regression models were calculated. In order to test the robustness of our models, we conducted multivariable adjustment considering common risk factors for vascular complications (sex, age, body mass index (BMI), peripheral artery disease, platelet count and time on Impella support). A *p* value < 0.05 was considered statistically significant. The statistical analyses were conducted with Stata/SE 16.1 (StataCorp, College Station, TX, USA).

## Results

### Patient and procedural characteristics

**Figure 1:**
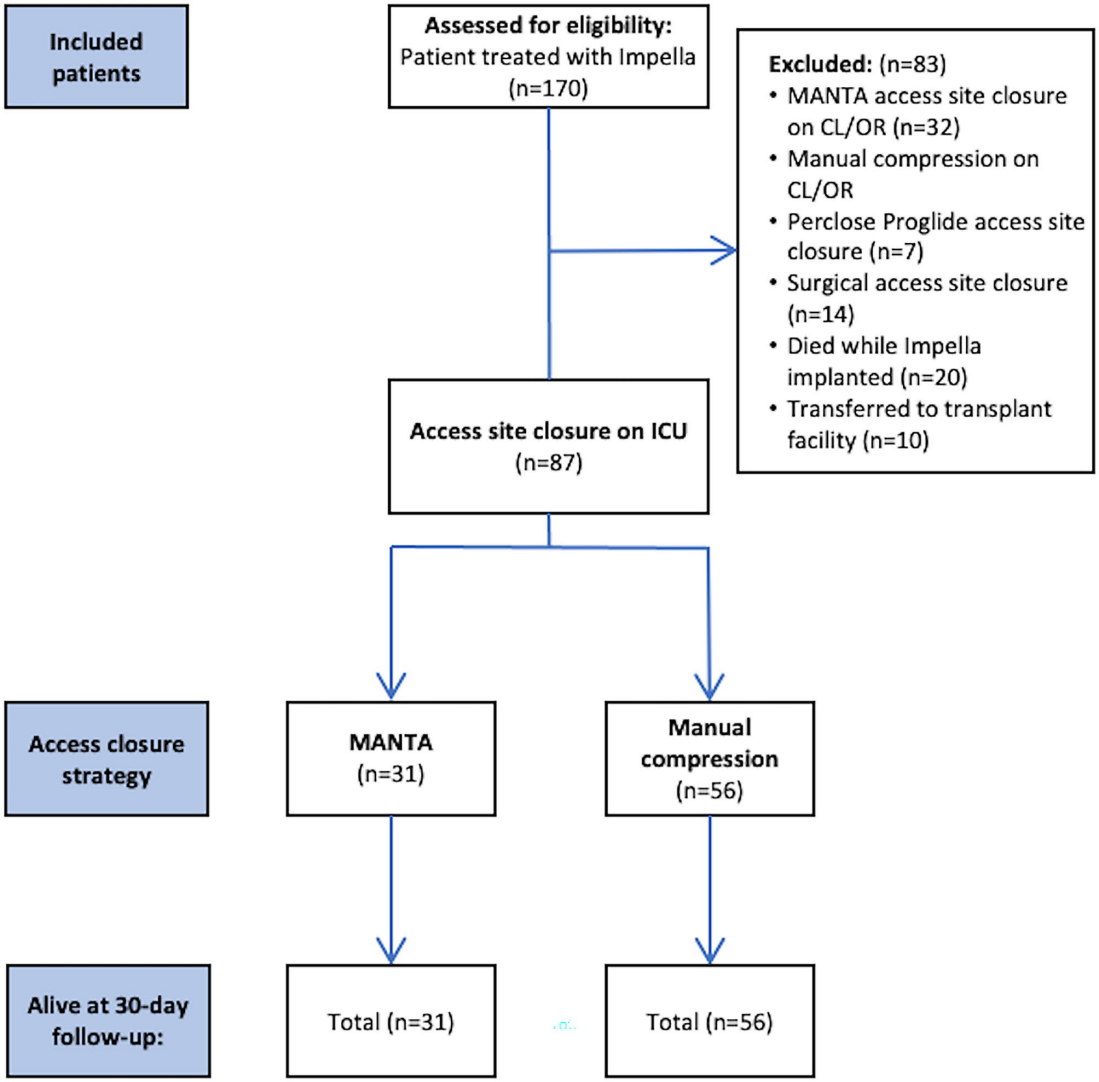
Study flow chart. MCS, mechanical circulatory support; ICU, intensive care unit; CL, catheterization laboratory; OR, operating room.

Overall, 170 patients required mechanical circulatory support with an Impella device between January 2017 and December 2020. Of those, in consecutive 87 patients (51%) the Impella device was removed directly on the ICU (Fig. 1). The mean age was 66.1 ± 10.6 years, 21 (24%) were females, the majority was recovering from CS (87%). Totally, 51 patients (58.6%) had initially presented with a STEMI. The baseline characteristics are depicted in Table 1. The MANTA device was used in 31 patients (35.6%) and manual compression was applied in 56 patients (64.4%). Of note, both groups were balanced in terms of comorbidities, including the presence of PAD (overall 13.8%), as highlighted in Table 1. Importantly, minimal femoral arterial diameter was similar in both groups (*p* = 0.26). The larger Impella CP was implanted in most patients (95.4%) and median hemodynamic support time was 39.8 h (IQR 23.6–68.9 h). However, hemodynamic support time was somewhat longer in the manual compression group than in the MANTA group (45.0 (IQR 28.5–75.1 h) versus 34.5 h (IQR 12.5–48.1 h); *p* = 0.036). The anatomical and procedural characteristics are presented in Table 2.

**Table 1:** Baseline demographics grouped according to the access closure strategy on ICU.

	Overall (n = 87)	MANTA device (n = 31)	Manual compression (n = 56)	P value*
Age (years)	66.1 ± 10.7	64.7 ± 9.1	66.9 ± 11.5	0.37
Female (%)	21 (24.1)	6 (19.4)	15 (26.8)	0.44
BMI (kg/m^2^)	26.1 ± 4.0	26.3 ± 4.6	26.0 ± 3.7	0.74
Cardiac arrest prior to admission	45 (51.7)	12 (38.7)	33 (58.9)	0.07
**Cause of CS**				0.53
STEMI	51 (58.6)	16 (51.6)	35 (62.5)	
NSTEMI/unstable AP	21 (24.1)	8 (25.8)	13 (23.2)	
No ACS	15 (17.2)	7 (22.6)	8 (14.3)	
**Comorbidities**				
Previous PCI	17 (19.5)	9 (29.0)	8 (14.3)	0.09
Previous MI	14 (16.1)	6 (19.4)	8 (14.3)	0.54
Previous CABG	8 (9.2)	2 (6.5)	6 (10.7)	0.51
PAD	12 (13.8)	6 (19.4)	6 (10.7)	0.26
Previous stroke/TIA	6 (6.9)	4 (12.9)	2 (3.6)	0.10
History of heart failure	3 (3.4)	0 (0)	3 (5.4)	0.19
Diabetes	27 (31.0)	14 (45.2)	13 (23.2)	0.039
Arterial hypertension	49 (56.3)	19 (61.3)	30 (53.6)	0.48
Dyslipidemia	36 (41.4)	14 (45.2)	22 (39.3)	0.59
Obesity (BMI > 30)	15 (17.2)	8 (25.8)	7 (12.5)	0.20
Current smoking	18 (20.7)	9 (29)	9 (16.1)	0.15
Chronic kidney disease^†^	11 (12.6)	4 (12.9)	7 (12.5)	0.95
Preexisting anemia	4 (4.6)	2 (6.5)	2 (3.6)	0.61
Initial LVEF	25 ± 8	25 ± 6	25 ± 9	0.77
LVEDP (mmHg)	32 ± 9	34 ± 9	32 ± 8	0.46
**Laboratory parameters**^**‡**^				
Peak lactate (mmol/L)	2.6 (1.7–4.5)	2.4 (1.6–3.2)	2.7 (1.9–5)	0.11
eGFR (CKD-EPI) (ml/min/1.73m^2^)	72 (52–91)	74.5 (63.5–91)	67 (48–90)	0.16
Peak troponin T (ng/L)	4323 (1547–12,384)	2901 (1547–9630)	4907 (1519–15,481)	0.29
Peak CK (IU/L)	3002 (811–6078)	1754 (779–5027)	3445 (992–6531)	0.29
Hemoglobin prior to closure (g/L)	97.8 ± 18.8	105 ± 20.1	93.8 ± 16.9	0.007
Thrombocytes (G/L)	160 ± 89	188 ± 104	145 ± 77	0.036
Anti-Xa level (U/mL)	0.47 (0.23–0.83)	0.54 (0.21–0.89)	0.47 (0.23–0.80)	0.73
**Type of intervention performed**				
PCI performed	75 (86.2)	26 (83.9)	49 (87.5)	0.64
CABG performed	3 (3.4)	2 (6.5)	1 (1.8)	0.25
**Antithrombotics**^**§**^				
Aspirin	78 (89.7)	26 (83.9)	52 (92.9)	0.19
Ticagrelor	56 (64.4)	17 (54.8)	39 (69.6)	0.16
Cangrelor	9 (10.3)	7 (22.6)	2 (3.6)	0.006
Prasugrel	1 (1.1)	0 (0)	1 (1.8)	0.45
Clopidogrel	9 (10.3)	3 (9.7)	6 (10.7)	0.87
GPIIbIIIa inhibitors	9 (10.3)	1 (3.2)	8 (14.3)	0.10
Unfractionated heparin	67 (77.0)	28 (90.3)	39 (69.6)	0.029
DOACs	2 (2.3)	0 (0)	2 (3.6)	0.28

Data are median (interquartile range) or number (percentage), as appropriate. AP, angina pectoris; BMI, body mass index; CS, cardiogenic shock; STEMI, ST-segment–elevation acute myocardial infarction; NSTEMI, non–ST-segment–elevation myocardial infarction; PCI, percutaneous coronary intervention; MI, myocardial infarction; PAD, peripheral arterial disease; CABG, coronary artery bypass grafting; TIA, transient ischemic attack; Hb, hemoglobin; LVEF, left ventricular ejection fraction; LVEDP, left ventricular end diastolic pressure; eGFR (CKD-EPI), estimated glomerular filtration rate (Chronic Kidney Disease Epidemiology Collaboration); CK, creatine kinase; DOAC, direct oral anticoagulant agents.

*P values were based on Fisher's exact, Man-Whitney-U test or student's t-test, as appropriate.

^†^Chronic kidney disease was defined as a preexisting eGFR < 45 ml/min/m^2^.

^‡^Values of laboratory parameters prior to access closure are represented (either first or peak, as appropriate).

^§^This comprised antithrombotics, which had been administered after Impella implantation and/or prior to device removal.

**Table 2:** Access and device-related characteristics.

	Overall (n = 87)	MANTA device (n = 31)	Manual compression (n = 56)	P value*
**Vessel characteristics**				
Minimal iliac artery diameter (mm)	7.8 ± 1.7	8.16 ± 1.8	7.5 ± 1.6	0.17
Minimal femoral artery diameter (mm)	7.2 ± 1.7	7.5 ± 2.0	7.0 ± 1.5	0.26
None to mild calcification	44 (50.6)	17 (54.8)	27 (48.2)	0.63
Moderate calcification	12 (13.8)	7 (22.6)	5 (8.9)	0.18
Severe calcification	8 (9.2)	2 (6.5)	6 (10.7)	0.33
Single site access	13 (14.9)	11 (35.5)	2 (3.6)	< 0.001
**Impella devices**				
2.5	4 (4.6)	0 (0)	4 (7.1)	0.12
CP/smart assist	83 (95.4)	31 (100)	52 (92.9)	0.12
**Impella implantation**^**†**^				
Easy	44 (50.6)	14 (45.2)	30 (53.6)	0.24
Some difficulties	28 (32.2)	12 (38.7)	16 (28.6)	0.47
Very challenging	8 (9.2)	4 (12.9)	4 (7.1)	0.44
Implantation under resuscitation	10 (11.5)	2 (6.5)	8 (14.3)	0.26
pLVAD support time (h)	39.8 (23.6–68.9)	34.5 (12.5–48.1)	45.0 (28.5–75.1)	0.036

Data are median (interquartile range) or number (percentage), as appropriate. pLVAD = percutaneous left ventricular assist device.

*P values were based on Man-Whitney-U test or t test as appropriate.

^†^This was defined based on the feedback from the interventionalist involved in the procedure and/or presence of procedure related complications.

### Access related bleeding and vascular complications

**Figure 2:**
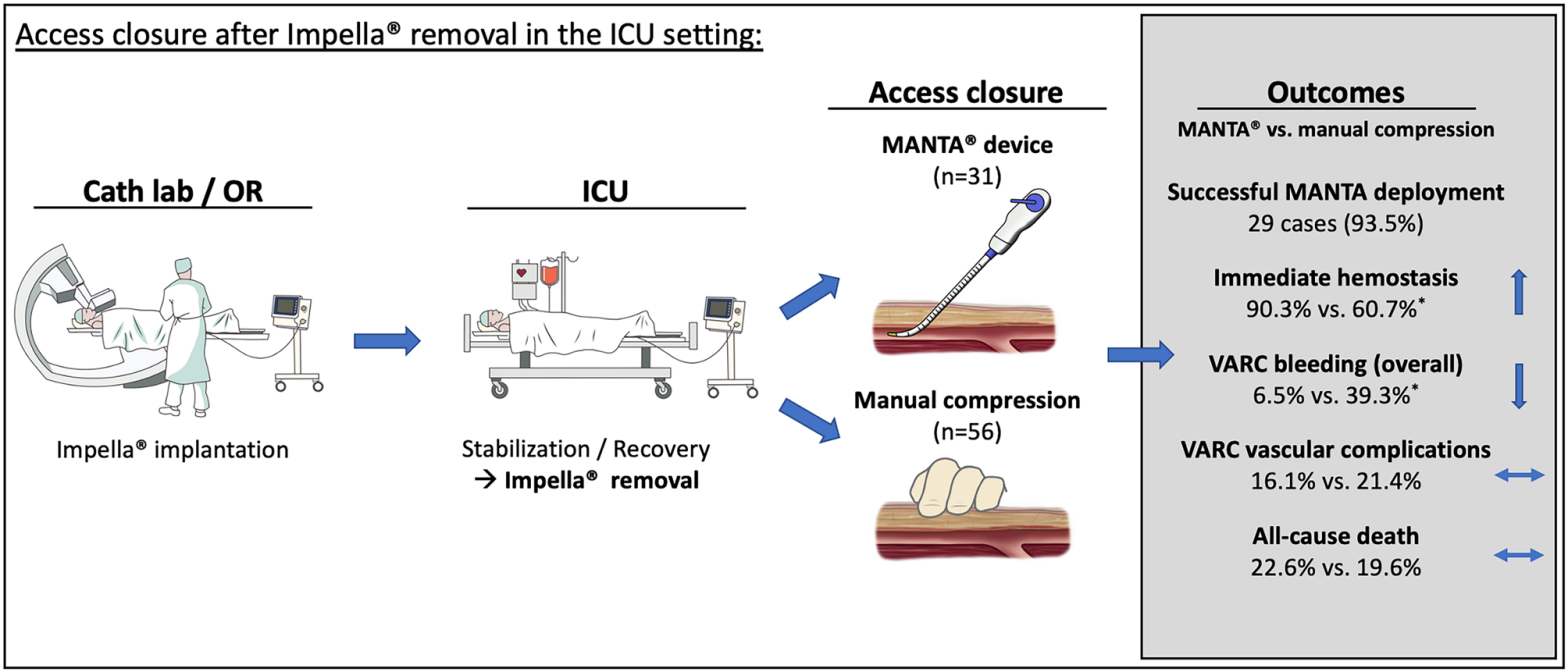
Access closure after Impella removal in the ICU setting. CU, intensive care unit; OR, operating room. *Indicates a statistically significant difference (p < 0.05).

Figure 2 summarizes the main results. Immediate hemostasis was significantly more often achieved with the MANTA device (90.3% versus 60.7%; odds ratio (OR) 5.76, 95% confidence interval (CI) 1.46–32.67; *p* = 0.005). However, overall strategy success rate was high (94.3%) and there was no significant difference between groups (*p* = 0.55). Overall, any access related complications were significantly lower in the MANTA compared to the manual compression group (19.4% versus 46.4%, OR 0.28, 95% CI 0.08–0.84; *p* = 0.012). Also, bleeding complications according to VARC-2 criteria were significantly lower in the MANTA group (6.5% versus 39.3%, OR 0.10, 95% 95% CI 0.01–0.5, *p* = 0.001). Regarding vascular complications, there was no difference between the two groups (16.1% versus 21.4%, OR 0.70, CI 0.17–2.47, *p* = 0.55). Access related outcomes are depicted in Table 3. The multivariable models are displayed in the Supplemental Tables 1 and 2. The narratives of major complications are presented in Supplemental Table 3. To establish a possible learning curve, we compared the outcomes of first 15 cases managed with the MANTA to the later cases, but we found no differences across all analyzed outcomes.

**Table 3:** Access related outcomes after Impella removal.

Access-related outcomes	Overall (n = 87)	MANTA device (n = 31)	Manual compression (n = 56)	OR (95% CI)*	P value^†^
Immediate hemostasis^‡^	62 (71.3)	28 (90.3)	34 (60.7)	5.76 (1.46–32.67)	0.0049
Successful MANTA deployment^§^		29 (93.5)	–	–	–
Strategy success^II^	82 (94.3)	29 (93.5)	53 (94.6)	0.54 (0.04–7.97)	0.55
**Any access-related adverse outcome**	32 (36.8)	6 (19.4)	26 (46.4)	0.276 (0.08–0.84)	0.012
**VARC bleeding**					
Overall	24 (27.6)	2 (6.5)	22 (39.3)	0.106 (0.01–0.50)	0.001
Minor bleeding (BARC 2)	16 (18.4)	1 (3.2)	15 (26.8)	0.091 (0.002–0.67)	0.006
Major bleeding (BARC 3a)	7 (8.0)	1 (3.2)	6 (10.7)	0.277 (0.005–2.49)	0.22
Life-threatening (BARC 3b/5)	1 (1.1)	0 (0)	1 (1.8)	-	0.45
**VARC vascular complications**					
Overall	17 (19.5)	5 (16.1)	12 (21.4)	0.70 (0.17–2.47)	0.55
Minor complications	3 (3.4)	2 (6.5)	1 (1.8)	3.79 (0.19–227.73)	0.25
Major complications	14 (16.1)	3 (9.7)	11 (19.6)	0.44 (0.07–1.88)	0.22
Urgent vascular surgery	7 (8.0)	3 (9.7)	4 (7.1)	1.39 (0.19–8.84)	0.67

Data are median (interquartile range) or number (percentage), as appropriate. BARC, Bleeding Academic Research Consortium; CI, confidence intervals; OR, odds ratio; VARC, Valve Academic Research Consortium.

*OR and CI was calculated using manual compression as control group.

^†^P values were based on Mann–Whitney-U test as appropriate.

^‡^Immediate hemostasis was defined as a state of no relevant bleeding or oozing with no additional medical action needed directly after access closure. Only 86 patients could be included for this, 1 patient in the manual compression group could not be evaluated.

^§^Successful MANTA deployment was defined as a correct release and placement of the MANTA toggle and plug and was evaluated at each closure by the responsible operator.

^II^Strategy success was achieved when there were no signs of bleeding within 30 min after Impella removal and, in case of MANTA usage, if there was correct deployment of the vascular closure device and no complications had occurred.

### Clinical outcomes at 30-days

No additional vascular complications occurred during the follow-up period. Importantly, we did not encounter any obvious major access site related infections following device removal and access closure. Nonetheless sepsis occurred in 10.3% of all patients (12.9% versus 8.9%, hazard ratio (HR) 0.67, 95% CI 0.18–2.52, *p* = 0.56), highlighted in Table 4. Also, there was no significant difference between all-cause mortality (22.6% versus 19.6%, HR 0.87, CI 0.34–2.26, *p* = 0.78). 30-day outcomes are reported in Table 4.

**Table 4:** Clinical outcomes at 30-day.

	Overall (n = 87)	MANTA device (n = 31)	Manual compression (n = 56)		P-value^†^
Any packed RBC transfusion	2 (0–5)	0 (0–4)	3 (1–5)		0.004
ICU stay (days)	5 (3–10)	4 (3–8)	6 (4–11)		0.14
Duration of hospital stay (days)	11 (7–20)	10.5 (7–18)	11 (7–20.5)		0.91
**Clinical outcomes at 30-days**				**HR (95%CI)**	
Minor bleeding (BARC 2)*	14 (16.1)	6 (19.4)	8 (14.3)	0.81 (0.23–2.86)	0.74
Major bleeding (BARC 3/5)*	37 (42.5)	13 (41.9)	24 (42.9)	0.87 (0.38–2.02)	0.75
New MI	5 (5.7)	4 (12.9)	1 (1.8)	0.13 (0.01–1.18)	0.07
Stroke/TIA	6 (6.9)	2 (6.5)	4 (7.1)	1.08 (0.2–5.91)	0.92
New heart failure	11 (12.6)	7 (22.6)	4 (7.1)	0.26 (0.06–1.03)	0.054
Repeat CS	7 (8.0)	3 (9.7)	4 (7.1)	0.71 (0.16–3.19)	0.65
Sepsis	9 (10.3)	4 (12.9)	5 (8.9)	0.68 (0.18–2.52)	0.56
Cardiovascular death	18 (20.7)	7 (22.6)	11 (19.6)	0.87 (0.34–2.26)	0.78
All-cause death	18 (20.7)	7 (22.6)	11 (19.6)	0.87 (0.34–2.26)	0.78

Data are median (interquartile range) or number (percentage), as appropriate. BARC, Bleeding Academic Research Consortium; CS, cardiogenic shock; HR, hazard ratio; ICU, intensive care unit; MI, myocardial infarction; RBC, red blood cells; TIA, transient ischemic attack.

*Any additional bleeding occurring within 30 days of follow-up.

^†^P values were based on Man-Whitney-U test or cox regression as appropriate.

## Discussion

Our study suggests that using the MANTA device for femoral arteriotomy closure after Impella device removal in the ICU setting is safe and effective. Compared to manual compression, we observed a lower incidence of access-related bleeding complications and similar vascular complication rates. Albeit earlier studies by our and other groups have indicated the efficacy and safety of the MANTA closure device after pLVAD removal, none of them investigated its use outside of a dedicated area such as the cardiac catheterization laboratory or the operating room^6,7^. To the best of our knowledge this is the first report describing the use of the MANTA device for large bore access-site management on the ICU.

As widely known, 30-day mortality in CS patients remains high (up to 50%) despite immediate treatment and hemodynamic support in the form of a pLVAD^12^. Patients recovering from CS and requiring mechanical support usually represent an extreme risk patient cohort, which is prone to complications. It is well known that bleeding is associated with a twofold increase in mortality rates in patients with acute myocardial infarction complicated by cardiogenic shock^13^. Additionally, intrahospital transportation has been shown to increase the complication rate of critically ill patients^14^. Consequently, adverse events related to poor femoral access management puts CS patients at great risk and the fashion as well as the locality of device removal need to be diligently planned and executed.

In line with previous reports, our study demonstrates that the MANTA closure device is safe and efficient for large-bore vessel closure^7,15^. Vascular complications after pLVAD removal remain a challenge, even for experienced operators. Despite of using the MANTA closure device on the ICU, vascular complication rates observed in our cohort (19.5%) were comparable to other studies describing outcomes after pLVAD removal in dedicated areas (4–17%)^7,16–19^. But one also needs to take in account that the majority of our patients underwent insertion of an Impella CP device in an emergent setting or even under ongoing resuscitation due to cardiogenic shock. Such a setting not just complicates the arteriotomy and device implantation, but it may be directly linked to a higher bleeding and vascular complication risk. Taken together, the rate of acute limb ischemia (2.3%) was relatively low in our cohort compared to prior studies analyzing outcomes after pLVAD placement (up to 13%)^17,18^.

When comparing the two strategies for access management, we observed no increased incidence of vascular complications after application of the MANTA device when compared to manual compression. On the contrary, the rate of access-related adverse outcome was twice as high in the manual compression group (Table 3). One also needs to take in account, that we did not identify any MANTA related access site or systemic infections in our cohort. Consequently, there was also no difference in the occurrence of sepsis or severe infections at 30-day follow-up between the two groups (Table 4). This is important, since patients in the critical care settings, and more so patients recovering from shock with organ dysfunction, are more susceptible to nosocomial infections^20^. Thus, our data may implicate that the risk for infectious complications with the delivery of the MANTA closure device in the ICU setting are very low, if carefully executed. However, physicians should also be aware that infectious complications related to the use of the MANTA device have been described^21^. Thus, this vascular closure device should not be deployed in patients with an untreated systemic or local infection (at or near the access site).

Although, other vascular closure device systems are available for large bore access site management, the MANTA closure device entails major advantages in the setting of CS patients. In contrast to percutaneous suture-mediated closure devices such as the Perclose ProGlide SMC System or the Prostar XL percutaneous vascular surgical system (Abbott Cardiovascular, Plymouth, MN), which may only be used in a pre-close fashion, post-procedural closure by the MANTA closure device allows immediate implantation of the pLVAD without delaying the potentially lifesaving PCI in these acutely ill patients.

Finally, device success rate in the MANTA group was high (94%). However, to achieve device success, pre-assessment by angiography or sonography of the femoral artery before pLVAD implantation appears key^7^. If feasible, we assess the arterial diameter, degree of calcification and the site of arterial puncture prior to Impella implantation. Visualization of the femoral artery can quickly be done before pLVAD implantation and certainly helps identifying patient with an unsuitable anatomy and therefore decreases the risk for vascular complications without delaying the procedure itself. Anyways, one needs to be aware that the application of the MANTA device requires some training and that there might be a learning curve, as highlighted in our recent publication^22^. Accordingly, physicians might only use this closure device in elective and well selected cases in the cath lab in the early phase of usage.

Manual compression for large-bore arterial access management after pLVAD removal is time-consuming, causes discomfort for patients and access-site bleeding control might not be achieved^23^. In contrast, the reduction in bleeding complications and faster mobilization after use of MANTA could potentially translate into shortened ICU stays and moreover mitigate the patients´ morbidity risk. However, this needs to be further investigated.

### Limitations

The results of the present study need to be interpreted in the light of the following limitations: Firstly, this was an observational, non-randomized single center study. Considering the small number of patients, the study was underpowered to identify predictors of worse outcome after MANTA device closure. Secondly, although the Impella removal and access site closure was performed by experienced interventional cardiologists, a learning curve might have had some impact on outcomes of patients in the MANTA group. Thirdly, standard operating procedures at our institution regarding the use of GP-IIb/IIIa-inhibitors have changed during the period of observation. Based on our experience and on published evidence showing the risk of GP-IIb/IIIa-inhibitors in the setting of large bore access, we completely stopped using these medications in patients with pLVADs.

## Conclusions

With respect to the growing number of patients treated with percutaneous left ventricular assist devices (pLVAD), for complex cardiac procedures, heart failure and shock, there is a need for enhanced strategies for access management. In this context, we studied the safety and efficacy of the novel MANTA closure device compared to manual compression for femoral arteriotomy closure after Impella removal in the ICU setting. Our data suggest that the application of the MANTA device can be safely used for this purpose in selected patients. Moreover, it seems to reduce access related bleedings without increasing the risk of vascular complications.

## Supplementary Information

Supplementary Information 1.

Supplementary Information 2.

Supplementary Information 3.
