# Supplementary material for: Manual compression versus MANTA device for access management after impella removal on the ICU
Source: Sci Rep. 2022 Aug 18;12:14060. doi: 10.1038/s41598-022-18184-x (PMC9388691; doi:10.1038/s41598-022-18184-x)
Supplement: Supplementary file 1 — Supplementary Information 1. [file 41598_2022_18184_MOESM1_ESM.docx]

**Supplemental table 1** Access related outcomes after Impella removal

| ***Access-related outcomes*** | ***Overall***  *(n=87)* | ***MANTA device***  *(n=31)* | ***Manual compression***  *(n=56)* | ***OR (95% CI)*** ^*^ | ***P-value****^†^* |
| --- | --- | --- | --- | --- | --- |
| Immediate hemostasis^‡^ | 62 (71.3) | 28 (90.3) | 34 (60.7) | 0.09 (0.01-0.54) | *0.008* |
| Successful MANTA deployment^§^ |  | 29 (93.5) | - | - | *-* |
| Strategy success ^II^ | 82 (94.3) | 29 (93.5) | 53 (94.6) | 0.34 (0.012-9.26) | *0.52* |
| **Any access-related adverse outcome** | 32 (36.8) | 6 (19.4) | 26 (46.4) | 6.601 (1.64-26.56) | *0.008* |
| *VARC Bleeding:* |  |  |  |  |  |
| Overall | 24 (27.6) | 2 (6.5) | 22 (39.3) | 15.78 (2.44-101.92) | *0.004* |
| Minor bleeding (BARC 2) | 16 (18.4) | 1 (3.2) | 15 (26.8) | 9.45 (1.05-84.77) | *0.045* |
| Major bleeding (BARC 3a) | 7 (8.0) | 1 (3.2) | 6 (10.7) | 9.54 (0.91-174.33) | *0.12* |
| Life-threatening (BARC 3b/5) | 1 (1.1) | 0 (0) | 1 (1.8) | - | *0.45* |
| *VARC vascular complications:* |  |  |  |  |  |
| Overall | 17 (19.5) | 5 (16.1) | 12 (21.4) | 2.44 (0.51-11.75) | *0.26* |
| Minor complications | 3 (3.4) | 2 (6.5) | 1 (1.8) | 0.52 (0.03-8.93) | *0.65* |
| Major complications | 14 (16.1) | 3 (9.7) | 11 (19.6) | 4.03 (0.59-27.34) | *0.15* |
| Urgent vascular surgery | 7 (8.0) | 3 (9.7) | 4 (7.1) | 1.30 (0.14-11.41) | *0.80* |

BARC = Bleeding Academic Research Consortium; CI = Confidence intervals; OR = Odds ratio; VARC = Valve Academic Research Consortium. Data are median (interquartile range) or number (percentage), as appropriate. Those models were adjusted for the following variables: sex, age, body mass index, peripheral artery disease, platelet count prior to access closure and Impella support time (hours).

^*^ OR and CI was calculated using manual compression as control group

^†^ P values were based on Mann-Whitney-U-test as appropriate.

^‡^ Immediate hemostasis was defined as a state of no relevant bleeding or oozing with no additional medical action needed directly after access closure. Only 86 patients could be included for this, 1 patient in the manual compression group could not be evaluated.

^§^ Successful MANTA deployment was defined as a correct release and placement of the MANTA toggle and plug and was evaluated at each closure by the responsible operator.

^II^ Strategy success was achieved when there were no signs of bleeding within 30 minutes after Impella removal and, in case of MANTA usage, if there was correct deployment of the vascular closure device and no complications had occurred.
