# Supplementary material for: Manual compression versus MANTA device for access management after impella removal on the ICU
Source: Sci Rep. 2022 Aug 18;12:14060. doi: 10.1038/s41598-022-18184-x (PMC9388691; doi:10.1038/s41598-022-18184-x)
Supplement: Supplementary file 2 — Supplementary Information 2. [file 41598_2022_18184_MOESM2_ESM.docx]

**Supplemental table 2** Clinical outcomes at 30-day

|  | ***Overall***  *(n=87)* | ***MANTA device***  *(n=31)* | ***Manual compression***  *(n=56)* |  | ***P-value****^†^* |
| --- | --- | --- | --- | --- | --- |
| Any packed RBC transfusion | 2 (0-5) | 0 (0-4) | 3 (1-5) |  | *0.004* |
| ICU stay (days) | 5 (3-10) | 4 (3-8) | 6 (4-11) |  | *0.14* |
| Duration of hospital stay (days) | 11 (7-20) | 10.5 (7-18) | 11 (7-20.5) |  | *0.91* |
| **Clinical outcomes at 30-days** |  |  |  | ***HR (95%CI)*** |  |
| Minor bleeding (BARC 2)^*^ | 14 (16.1) | 6 (19.4) | 8 (14.3) | 0.81 (0.23-2.86) | *0.74* |
| Major bleeding (BARC 3/ 5)^*^ | 37 (42.5) | 13 (41.9) | 24 (42.9) | 0.87 (0.38-2.02) | *0.75* |
| New MI | 5 (5.7) | 4 (12.9) | 1 (1.8) | 0.13 (0.01-1.18) | *0.07* |
| Stroke / TIA | 6 (6.9) | 2 (6.5) | 4 (7.1) | 1.08 (0.2-5.91) | *0.92* |
| New heart failure | 11 (12.6) | 7 (22.6) | 4 (7.1) | 0.26 (0.06-1.03) | *0.054* |
| Repeat CS | 7 (8.0) | 3 (9.7) | 4 (7.1) | 0.71 (0.16-3.19) | *0.65* |
| Sepsis | 9 (10.3) | 4 (12.9) | 5 (8.9) | 0.68 (0.18-2.52) | *0.56* |
| Cardiovascular death | 18 (20.7) | 7 (22.6) | 11 (19.6) | 0.87 (0.34-2.26) | *0.78* |
| All-cause death | 18 (20.7) | 7 (22.6) | 11 (19.6) | 0.87 (0.34-2.26) | *0.78* |

Data are median (interquartile range) or number (percentage), as appropriate. BARC = Bleeding Academic Research Consortium; CS = Cardiogenic shock; HR = Hazard ratio; ICU = Intensive care unit; MI = Myocardial Infarction; RBC = Red blood cells; TIA = Transient ischemic attack

^*^ Any additional bleeding occurring within 30 days of follow-up

^†^ P values were based on Man-Whitney-U test or cox regression as appropriate.

^‡^ Those models were adjusted for the following variables: sex, age, body mass index, peripheral artery disease, platelet count prior to access closure and Impella support time (hours).
