# Supplementary material for: Manual compression versus MANTA device for access management after impella removal on the ICU
Source: Sci Rep. 2022 Aug 18;12:14060. doi: 10.1038/s41598-022-18184-x (PMC9388691; doi:10.1038/s41598-022-18184-x)
Supplement: Supplementary file 3 — Supplementary Information 3. [file 41598_2022_18184_MOESM3_ESM.docx]

**Supplemental Table 3** Narratives of major complications

| ***Case no.*** *^*^* | ***Type of CS*** | ***SCAI***  ***class*** | ***Known PAD*** | ***Vascular***  ***complication*** | ***Bleeding (VARC)*** | ***Description*** |
| --- | --- | --- | --- | --- | --- | --- |
| **MANTA^®^ device cohort** | |  |  |  |  |  |
| #5 | Ischemic (NSTEMI) | D | Yes | Major | None | ALI due to thrombus formation after Impella^®^ removal, finally requiring by surgical thrombectomy. |
| #9 | Ischemic (STEMI) | C | None | Major | None | ALI due to thromboembolism requiring vascular intervention (thrombus aspiration and thrombolysis). |
| #17 | Ischemic (STEMI) | D | None | Minor | None | Pseudoaneurysm development requiring surgical repair. |
| #21 | Ischemic (STEMI) | E | Yes | Major | Major | Rupture of aorto-bifemoral graft (Maquet Hemagard^®^) after Impella^®^ removal and failed MANTA^®^ device, requiring. urgent graft and arterial repair (including thrombectomy and surgical MANTA^®^ device removal). |
| #25 | Ischemic (STEMI) | E | None | Minor | None | Closure failure due to upside-down implantation of MANTA^®^ device and need of a second MANTA^®^ device. |
| #29 | Non-ischemic (Takotsubo) | D | None | None | Minor  (BARC 2) | After Impella^®^ removal and MANTA^®^ deployment minor bleeding managed with prolonged compression and halting of antithrombotics (unfractionated heparin). |
| **Manual compression cohort** | | | |  |  |  |
| #1 | Ischemic (STEMI) | D | None | Major | Major | Retroperitoneal bleeding after Impella^®^ removal treated with 2 RBC transfusions and prolonged compression (including Femostop^®^ therapy). |
| #3 | Ischemic (NSTEMI) | C | None | Major | None | Critical leg ischemia with no reperfusion option due to poor vascular and overall health condition. |
| #4 | Ischemic (STEMI) | E | None | Major | Major | Bleeding requiring 2 RBC transfusions and surgical repair of pseudoaneurysm. |
| #9 | Non-ischemic | E | None | Major | Major | Bleeding requiring 2 RBC transfusions. |
| #13 | Non-ischemic | E | None | Major | Life threatening | Bleeding at access site requiring 4 RBC transfusions. |
| #20 | Ischemic (STEMI) | E | None | None | Minor | Bleeding requiring treatment with 1 RBC transfusion. |
| #23 | Non-ischemic | E | None | Minor | Minor | Access related bleeding treated with prolonged compression (Femostop^®^); skin necrosis after prolonged compression. |
| #25 | Ischemic (NSTEMI) | D | None | Major | Major | Access related bleeding (requiring 2 RBC tranfusions). |
| #27 | Ischemic (STEMI) | D | None | Major | Major | Bleeding with 3 RBC, pseudoaneurysm requiring urgent surgical repair. |
| #29 | Ischemic (STEMI) | D | None | None | Minor | Persistent bleeding treated with prolonged compression (Femostop^®^). |
| #31 | Ischemic (STEMI) | D | None | Major | None | Leg ischemia and prolonged hypesthesia after compression using Femostop^®^. Conservatively managed (anticoagulation). |
| #40 | Ischemic (NSTEMI) | B | Yes | Major | Major | Access related bleeding treated with 2 RBC transfusions and major vagal reaction triggered by manual compression. |
| #41 | Ischemic (STEMI) | C | None | Major | None | Leg ischemia due to thrombus formation requiring urgent surgical repair. |
| #56 | Ischemic (NSTEMI) | C | None | Major | None | Leg ischemia due to distal thromboembolism treated with urgent thrombectomy and PTA. |

ALI = Acute limb ischemia; BARC = Bleeding Academic Research Consortium; CS = Cardiogenic shock; FFP = Fresh frozen plasma; Hb = Hemoglobin (g/dl); ICU = Intensive care unit; NSTEMI = Non-ST-segment elevation myocardial infarction; PAD = Peripheral arterial disease; PTA = Percutaneous transluminal angioplasty; RBC = Red blood cells; SCAI = Society of Cardiovascular Angiography and Intervention; STEMI = ST-segment myocardial infarction; VARC = Valve Academic Research Consortium.

*^*^* Case number refers to procedure using MANTA^®^ for access closure
